# Spondyloarthritis recognition for primary care: A simplified diagnostic and referral pathway for general physicians

**DOI:** 10.1515/rir-2025-0030

**Published:** 2025-12-27

**Authors:** Anum Khan, Babur Salim, Shahida Perveen, Samina Mushtaq, Saba Samreen, Haris Gul

**Affiliations:** Consultant Rheumatologist, Bahria International Hospital, Rawalpindi, Pakistan; Rheumatology department Fauji Foundation Hospital, Rawalpindi, Pakistan; Rheumatology department, Foundation University School of Health Sciences, Fauji Foundation Hospital, Rawalpindi, Pakistan; Capital Hospital, CDA Islamabad, Pakistan

Dear Editor,

Spondyloarthritis (SpA) represents a group of interrelated inflammatory conditions whose diagnostic challenges far exceed their textbook definitions. SpA’s true clinical burden lies in its insidious onset and remarkable phenotypic variability.^[1]^

Despite advances in classification criteria, spondyloarthritis (SpA) is still plagued by diagnostic delays averaging 5–7 years^[2]^ —even longer in women and non-radiographic cases. Diagnostic delay leads to irreversible joint damage, spinal fusion, and functional disability, while early intervention preserves mobility and quality of life. Untreated inflammation also increases risks of osteoporosis, cardiovascular disease, and chronic pain. The prolonged diagnosis time reflects several key barriers:

## Barriers to Early Recognition

Subtle early symptoms: Inflammatory back pain (IBP) is often misattributed to mechanical causes.

Gender disparities: Women more often present with atypical features (*e.g*., cervical pain, fatigue, predominant peripheral symptoms) and slower radiographic progression, leading to underdiagnosis.^[2]^

A global shortage of rheumatologists means most SpA cases initially present to general practitioners, many of whom lack specialized training in detecting early inflammatory musculoskeletal disease.

### Overlap with Fibromyalgia: A Diagnostic Pitfall

The clinical presentations of spondyloarthritis and fibromyalgia often overlap, particularly about pain characteristics, fatigue patterns, and sleep dysfunction, creating diagnostic challenges.

Up to 20% of SpA patients develop secondary fibromyalgia,^[3]^ complicating assessments due to shared features.

### Complex Labs/ Imaging Findings in SpA Diagnosis

A) C-reactive protein (CRP)/Erythrocyte sedimentation rate (ESR): Elevated in only 35%–50% of active cases, limiting reliability for disease monitoring.^[4]^

B) Pelvic X-rays: Detect sacroiliitis (modified New York criteria^[5])^ in ~50% of patients, usually after 2–5 years of symptoms—missing early disease.^[6]^

C) Magnetic resonance imaging (MRI, sacroiliac joints): (a) Gold standard for early detection (70% show inflammation despite normal X-rays ^[6]^). (b) Challenges: Subtle findings, excessive cost, and nonspecific changes (*e.g*., degenerative *vs*. inflammatory). Spine MRI often reveals incidental abnormalities, complicating interpretation.

D) Human leukocyte antigen (HLA)-B27: Positive in 75%–90% of cases but limited by false negatives, cost, and access barriers in resource-poor settings.^[7]^

### Resource limitations

In low-income regions, restricted access to MRI and HLA-B27 testing forces reliance on clinical judgment alone.

## A Pragmatic Solution: The General Physician Referral Algorithm

**Figure 1:**
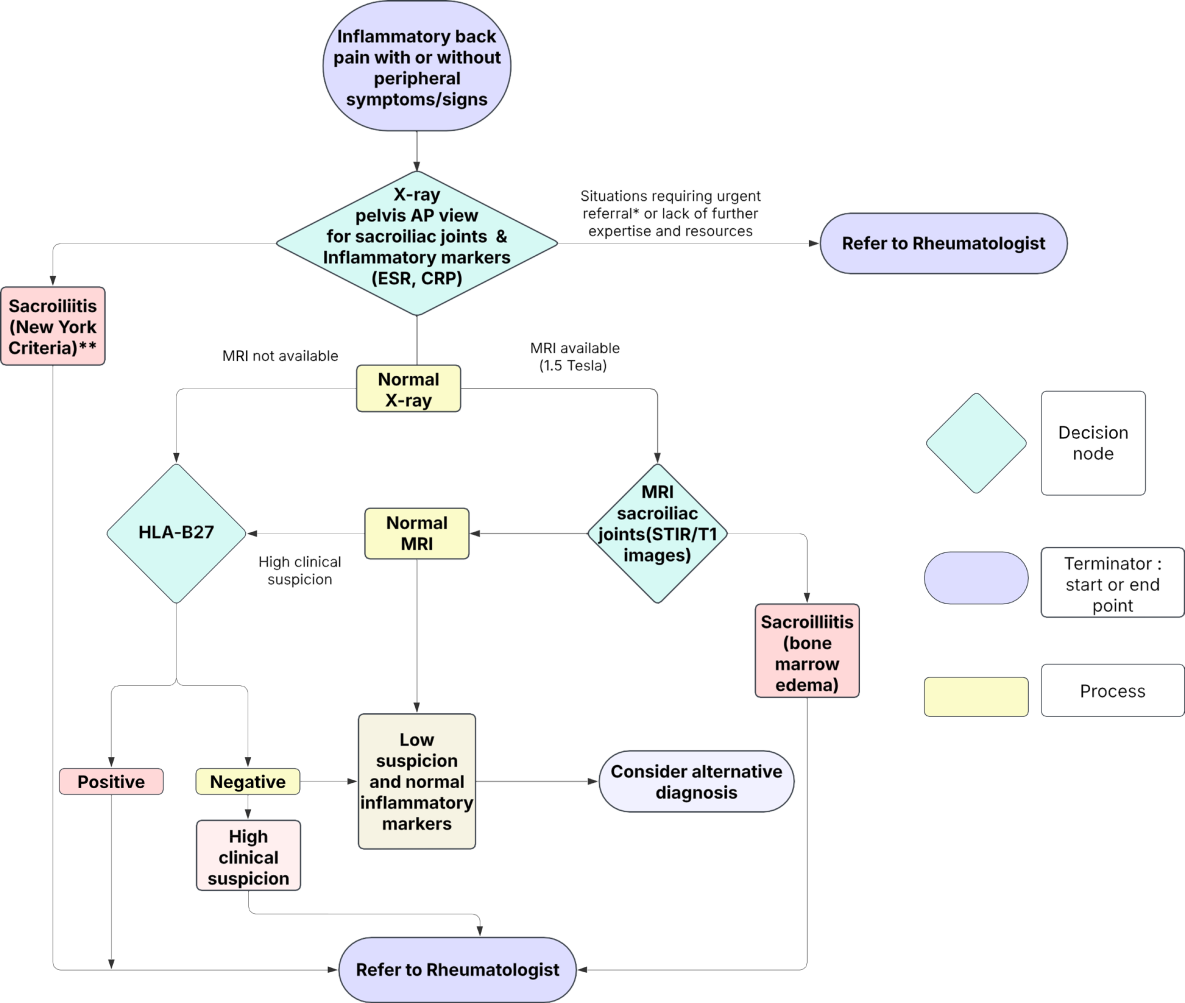
SpA referral Algorithm. *Uveitis, peripheral arthritis, severe or progressive axial disease and severe functional impairment, dactylitis, lBD-associated SpA, psoriatic arthritis with severe skin or ioint involvement, suspected juvenile spondyloarthritis; **New York criteria for sacroilitis: Grade 2 bilaterally or Grade 3 unilaterally is diagnostic (reference in text).

To bridge these diagnostic gaps, we developed a tiered primary care protocol (Figure 1) that diverges from specialty-centric Assessment of Spondyloarthritis International Society (ASAS) criteria^[8]^ by prioritizing pragmatic, resource-conscious decision-making. Our algorithm, refined through rheumatologist consensus in Rawalpindi/Islamabad, operationalizes SpA identification through:

### Clinical Triage

A) High-probability features: Inflammatory back pain (age < 45, > 3 months), enthesitis (Achilles/plantar), peripheral arthritis, dactylitis, or family history.

B) Red flags: Fever, weight loss, or neurological deficits (neurosurgical referral).

### Staged Diagnostics

A) First-line: CRP/ESR + pelvic X-ray (NY criteria ^[5]^).

B) Second-line: MRI SI joints (if X-ray negative) or HLA-B27 (if MRI unavailable).

### Interim Management

A) Nonsteroidal antiinflammatory drugs (NSAIDs, with response documentation).

B) Physiotherapy and smoking cessation counselling.

C) Education on early intervention benefits.

D) Urgent rheumatology referral in these situations: a), Acute anterior uveitis; b), Peripheral arthritis or progressive axial disease; c), Poor NSAID response with elevated markers.

## Expected Impact

In conclusion, the proposed referral algorithm provides a structured, pragmatic framework for general practitioners in resource-limited settings to identify patients with high-risk features of SpA. By demystifying complex diagnostic criteria and offering a clear, tiered pathway for investigation and referral, this tool empowers primary care physicians to become effective first-line screeners. Its implementation promises to significantly shorten diagnostic delays, optimize the use of costly diagnostics like MRI, and ensure that patients who need it most receive expedited specialist care. Ultimately, this algorithm is a vital step towards bridging the gap between primary and specialty care, mitigating the long-term disability and systemic complications associated with undiagnosed SpA.

## References

[1] Walsh JA, Magrey M (2021). Clinical Manifestations and Diagnosis of Axial Spondyloarthritis. J Clin Rheumatol.

[2] Zhao SS, Pittam B, Harrison NL (2021). Diagnostic delay in axial spondyloarthritis: a systematic review and meta-analysis. Rheumatology (Oxford).

[3] Olfa S, Bouden S, Sahli M (2023). Fibromyalgia in Spondyloarthritis: Prevalence and Effect on Disease Activity and Treatment. Curr Rheumatol Rev.

[4] Jurkowski M, Jeong S, Brent LH (2021). Axial Spondyloarthritis: Clinical Features, Classification, and Treatment. JSM Arthritis.

[5] Sieper J, Braun J (2009). Clinician’s Manual on Ankylosing Spondylitis.

[6] Nicolaes J, Tselenti E, Aouad T (2025). Performance analysis of a deep-learning algorithm to detect the presence of inflammation in MRI of sacroiliac joints in patients with axial spondyloarthritis. Ann Rheum Dis.

[7] Mariani FM, Alunno A, Di Ruscio E (2023). Human Leukocyte Antigen B*27-Negative Spondyloarthritis: Clinical, Serological, and Radiological Features of a Single-Center Cohort. Diagnostics (Basel).

[8] Sepriano A, Rubio R, Ramiro S (2017). Performance of the ASAS classification criteria for axial and peripheral spondyloarthritis: a systematic literature review and meta-analysis. Ann Rheum Dis.

